# Social Networks, Trust, and Disaster-Risk Perceptions of Rural Residents in a Multi-Disaster Environment: Evidence from Sichuan, China

**DOI:** 10.3390/ijerph18042106

**Published:** 2021-02-22

**Authors:** Kaijing Xue, Shili Guo, Yi Liu, Shaoquan Liu, Dingde Xu

**Affiliations:** 1 Institute of Mountain Hazards and Environment, Chinese Academy of Sciences, #9, Block 4, Renminnan Road, Chengdu 610041, China; kaijingxue@imde.ac.cn; 2 University of Chinese Academy of Sciences, No. 19A Yuquan Road, Beijing 100049, China; 3 China Western Economic Research Center, Southwestern University of Finance and Economics, Chengdu 610074, China; guoshili@swufe.edu.cn; 4 College of Management of Sichuan Agricultural University, Chengdu 611130, China; lyx1@stu.sicau.edu.cn; 5 Sichuan Center for Rural Development Research, College of Management, Sichuan Agricultural University, 211 Huimin Road, Wenjiang District, Chengdu 611130, China

**Keywords:** social networks, trust, risk perception, multiple disasters, China

## Abstract

Individual perception of disaster risk is not only the product of individual factors, but also the product of social interactions. However, few studies have empirically explored the correlations between rural residents’ flat social networks, trust in pyramidal channels, and disaster-risk perceptions. Taking Sichuan Province—a typical disaster-prone province in China—as an example and using data from 327 rural households in mountainous areas threatened by multiple disasters, this paper measured the level of participants’ disaster-risk perception in the four dimensions of possibility, threat, self-efficacy, and response efficacy. Then, the ordinary least squares method was applied to probe the correlations between social networks, trust, and residents’ disaster-risk perception. The results revealed four main findings. (1) Compared with scores relating to comprehensive disaster-risk perception, participants had lower perception scores relating to possibility and threat, and higher perception scores relating to self-efficacy and response efficacy. (2) The carrier characteristics of their social networks significantly affected rural residents’ perceived levels of disaster risk, while the background characteristics did not. (3) Different dimensions of trust had distinct effects on rural residents’ disaster-risk perceptions. (4) Compared with social network variables, trust was more closely related to the perceived level of disaster risks, which was especially reflected in the impact on self-efficacy, response efficacy, and comprehensive perception. The findings of this study deepen understanding of the relationship between social networks, trust, and disaster-risk perceptions of rural residents in mountainous areas threatened by multiple disasters, providing enlightenment for building resilient disaster-prevention systems in the community.

## 1. Introduction

Natural disasters are events in which natural changes exceed what can be borne by humans, thereby causing harm to human society and the economy [1]. Natural disasters mainly include geophysical disasters—such as earthquakes and volcanoes—and disasters caused by weather or climate—such as floods, storms, and landslides. In recent years, with changes in global climate and increases in the scope and intensity of human activities, the frequency and degree of harm of various natural disasters have risen significantly, which has had far-reaching impacts on global economic and social development. In 2019, nearly 1900 natural disasters displaced 24.9 million people in 140 countries and regions, causing an estimated 137 billion dollars in economic losses according to the Internal Displacement Monitoring Centre and the Swiss Re Institute [2,3]. It is worth noting that Asia was among the most affected regions, both in terms of the number of people affected and the economic losses caused.

Mountainous areas, in addition to being regions with frequent natural disasters, are also characterized by chain reactions and mass occurrence of disasters. Residents in mountainous areas, especially rural residents, live in scattered communities with weak economic foundations and insufficient awareness of disaster prevention, all of which lead to more severe disaster threats [4,5]. China is a mountainous country: mountains account for 69% of the total land area and 45% of the population live in mountainous areas [6,7,8]. China is also a disaster-prone country [9]. From 2010 to 2018, about 244 million people were affected by natural disasters in China, resulting in direct economic losses of about 3520.4 billion yuan [10]. These disasters included 129 earthquakes with a magnitude of five or above and 117,299 geological disasters such as landslides and debris flows.

In regions where multiple disaster risks coexist and there is a risk of serious harm, effective risk management has become a challenge for governments and academia. However, previous studies on disaster-risk management have primarily focused on single types of disasters such as earthquakes, floods, or landslides [11,12,13]; there has been little attention paid to situations where the risk of multiple disasters coexists. Furthermore, the research areas were mainly in developed countries such as the USA and in Europe [14,15,16], with relatively few in developing countries or in Asia.

Many empirical studies have shown that residents’ risk perceptions will prompt them to take active risk mitigation actions [17,18]. For example, Miceli et al. [19] investigated the relationship between flood risk perceptions and prevention preparedness of residents in the northern Italian mountains, and found that residents’ anxiety and perceptions of flood-risk possibility were positively correlated with prevention preparedness. Xu et al. [20] explored the correlation between landslide risk perception and prevention behavior and found that residents’ perceived levels of the possibility and threat had significant and positive effects on active disaster preparedness. As disaster-risk perception plays an important role in the construction of disaster prevention and reduction systems at the family level, theories relating to disaster-risk perception are attracting increasing attention from scholars and managers. Disaster-risk perception evaluates the levels of an individual’s impression and awareness of disaster risks. Based on different goals, scholars have measured disaster-risk perceptions from different dimensions, including the possibility, impact, severity, controllability, and fear of disaster risks [19,21,22,23]. However, as the final point of disaster-risk management is the level of disaster prevention and mitigation activity, considering only how people feel about the disaster event itself fails to closely combine residents’ risk perceptions with corresponding adaptive behaviors.

On the basis of previous disaster-risk perception studies, some scholars have undertaken additional exploration of the relationships among residents’ self-efficacy, response efficacy, risk perceptions, and individual adaptive actions [17,24,25,26]. In essence, the intrinsic meaning of residents’ self-efficacy and response efficacy for disaster risks is the perception evaluation that they use to solve and deal with the disaster threats, respectively [27,28]. Therefore, in a broad sense, residents’ self-efficacy and response efficacy for disaster risks also belong to residents’ perceptions of disaster risks. Theoretically, it is feasible to integrate these two dimensions and disaster-risk perceptions into generalized disaster-risk perceptions. This study considered that generalized disaster-risk perception should include the perception evaluation of the disaster risk event itself as well as the degree of mitigation that can be achieved. However, few scholars have explored the integration of these two aspects; therefore, there is still a need to measure residents’ levels of generalized disaster-risk perception so that these can be more closely connected to residents’ adaptive actions.

In terms of the factors that influence residents’ disaster-risk perceptions, many studies have focused on the impact of socio-economic factors of individuals and families—such as gender, age, education, duration of residence, family population, etc.; disaster experience—including whether they had experienced a disaster, the number of times of disaster experience, etc.; and response preparation—such as building reinforcement, disaster insurance, etc. [20,29,30,31,32,33]. Social networks are a form of social resource that can provide people with social support [34]. Especially in mountainous rural areas with relatively isolated information, social networks can become the carrier of disaster-risk information, affecting individuals’ thinking and judgment regarding disaster risks, and thus continuously adjusting residents’ perceptions of disaster risks [35,36,37,38]. Therefore, residents’ perceptions of disaster risks are not only the product of personal factors, but also the product of interpersonal and social interaction processes. However, previous academic research has mostly analyzed social networks as a part of social capital and social support, and there has been little empirical research on the correlation between social networks and disaster-risk perceptions [39,40]; that is, previous research approaches have failed to characterize social networks as carriers. Therefore, the challenge of measuring social networks reasonably, according to their characteristics, and then determining relationships between social networks and disaster-risk perceptions is worth exploring. In addition, inside the community, residents get some services from the community management organization and are also bound by it to a certain extent. Previous studies have shown that residents’ trust in community management organizations is a key factor affecting their perceptions of disaster risks [32,40,41]. In fact, the information and safeguard measures confirmed and released by management organizations generally come from higher-level formal organizations with high authority and credibility, indicating that residents’ trust in community management organizations is trust in formal pyramidal channels [42]. However, the information and the support contained in social networks are more accessible but less exact, which shows the informal flat characteristics of social networks [43]. Accordingly, the effects of these two different types of channels on residents’ perceptions of disaster risks are another question worth exploring.

In this context, taking Sichuan Province—a typical disaster-prone province in China—as an example, and selecting residents in mountainous areas threatened by earthquakes, landslides, mountain torrents, and other disasters as the research object, this study measured the level of the interviewees’ disaster-risk perception in terms of the four dimensions of possibility, threat, self-efficacy, and response efficacy. Furthermore, the ordinary least squares method was used to probe the correlations and differences between social networks, trust in community management organizations, and residents’ disaster-risk perceptions to enrich the relevant research and provide reference for the government to formulate disaster prevention and mitigation measures.

## 2. Material and Methods

### 2.1. Research Area

Located in southwest China, Sichuan Province is dominated by hills and mountains which account for about 90% of the total area [44,45,46]. Sichuan is a typical disaster-prone province in China. Apart from earthquakes, there are also geological disasters such as landslides and mud-rock flows. From 2008 to 2018, 19 earthquakes of magnitude five or above occurred in Sichuan province, causing a total of 460,000 casualties and 856.8 billion yuan of direct economic losses, accounting for 12.34% of the total number of earthquake disasters of magnitude five and above nationwide, 95.04% of the total number of disaster casualties nationwide, and 83.13% of direct economic losses nationwide caused by disasters. Additionally, a total of 18,518 geological disasters such as landslides and mud-rock flows have occurred in Sichuan Province, causing 1390 casualties and eight billion yuan of direct economic losses; these accounted for 11.99% of all geological disasters, 16.45% of all casualties, and 13.60% of direct economic losses nationwide [10]. Of these disasters, the Wenchuan earthquake on 12 May 2008 (8 on the Richter scale) and the Lushan earthquake on 20 April 2013 (7 on the Richter scale) caused huge casualties and economic losses to local residents [23,47]. Considering the non-negligible impact of earthquake disasters on residents in multi-disaster environments, this study selected the mountainous areas hit by the Wenchuan and Lushan earthquakes as the representative research areas within Sichuan Province.

### 2.2. Data Sources

**Figure 1:**
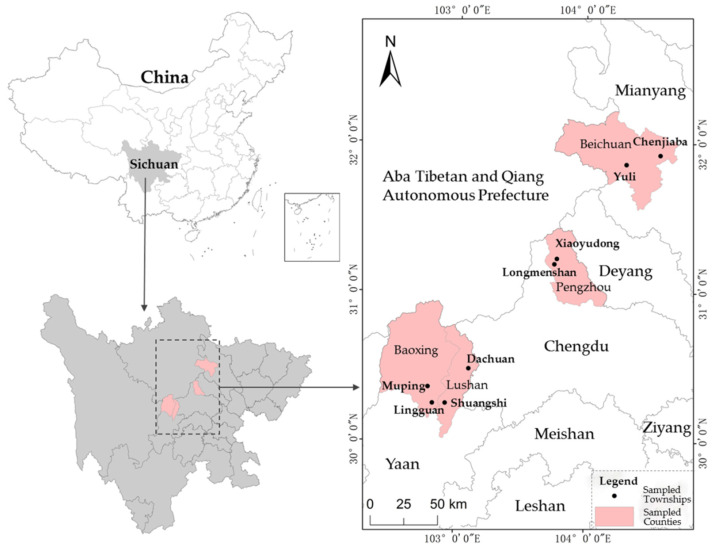
Distribution of sample counties and townships.

The data applied in this paper are primarily from a questionnaire survey conducted in July 2019 by the research group in the mountainous areas affected by the Wenchuan and Lushan earthquakes. This survey mainly investigated rural residents’ sustainable livelihoods, disaster-risk perceptions, disaster-avoidance behaviors, and the construction of resilient disaster-prevention systems in the community. The survey method was a face-to-face interview of residents for about 90 min. To ensure the representativeness of the selected samples, stratified sampling and then equal probability random sampling were used to determine the research samples [48]. First, considering that the sampled counties should come from areas affected by Wenchuan and Lushan earthquakes and that at least two counties with significant differences in economic development should be selected from the same disaster area, Beichuan County and Pengzhou City (Pengzhou City is a county-level city) were selected as sample counties from 10 counties stricken by the Wenchuan Earthquake, and Baoxing County and Lushan County were selected as sample counties from six counties hit by the Lushan Earthquake. Second, according to differences in the level of economic status within a county, the distance from the center of the county and the severity of the disaster, two sample townships were chosen from each sample county. In this way, a total of eight sample towns were obtained. Third, the villages in each sample town were divided into two groups according to the number of threatened people, the difference in economic development level, and the distance from the center of the town, and one village was randomly chosen from each group. By these means, 16 sample villages were obtained in all. Finally, in each sample village, 20–23 rural households were randomly chosen with reference to a roster and random number chart [33,34,49]. According to the survey, there are a total of 1145 disaster-threatened households in 16 selected villages. Further, based on the above process, a total of 327 valid questionnaires were gained from 16 villages in 8 townships in 4 counties. The spatial locations of the sample counties and townships are shown in Figure 1.

### 2.3. Variables and Methods

#### 2.3.1. Selection and Definition of Model Variables

(1) Dependent variables

The dependent variables in this paper were rural residents’ levels of disaster-risk perception. As mentioned above, the disaster-risk perception explored in this study was generalized, including the perception evaluation of the disaster-risk event itself and the degree of mitigation that could be achieved. Referring to the measurement methods of disaster-risk perceptions in existing literature [15,17,19,21,22,50], and combining with the data characteristics of acquired questionnaires, this paper mainly categorized entries in terms of four dimensions of disaster-risk perception—possibility, threat, self-efficacy, and response efficacy—to measure the generalized disaster-risk perception. The specific entries can be seen in Table 1. It is worth noting that, according to the survey, the types of disasters threatening residents in the study area mainly included earthquakes, landslides, debris flows, and mountain torrents. Therefore, this study relates particularly to these four types of disaster, generically. In addition, since many studies have shown that the response measures for different types of disasters are distinct, and residents’ perceptions of the effectiveness of different response measures are also distinct [51,52,53], disaster mitigation behaviors should be suitable for the four types of disasters and choose a clear response behavior, as far as possible, to measure response efficacy. Several studies have shown that evacuation—a common behavior to avoid disasters—can effectively reduce the adverse impact of disasters on residents [33,54,55,56] and can be well adapted to a variety of disaster types. Therefore, the response efficacy in this study specifically refers to the degree of disaster-threat mitigation by evacuation.

The specific measurement process was as follows. An internal consistency test was carried out on the entries characterizing residents’ perceptions of disaster risks, with results showing that Cronbach α values corresponding to the possibility, threat, self-efficacy, response efficacy, and comprehensive perception of disaster risks were all greater than 0.60 (0.69, 0.63, 0.66, 0.81, and 0.65, respectively). This indicated that the entries were internally consistent. Then, factor analysis was used to reduce dimensionality of the disaster-risk perception entries, and four dimensions of probability, threat, self-efficacy, and response efficacy were obtained. Among these, the Kaiser–Meyer–Olkin value corresponding to factor analysis was 0.72, the P value of the Bartlett test for sphericity was 0.000 (less than 0.001), and the cumulative variance contribution rate of the four dimensions was 64.62%; all of these results indicated that the results of the factor analysis were reasonable (see Table 2 for details). Then, the min-max standardization method was adopted to convert the four-dimensional scores obtained through factor analysis into a centesimal system, according to Equation (1). Finally, the ratio of the contribution rate of single dimensional variance to the contribution rate of cumulative variance was used as the weighting to calculate residents’ comprehensive perception of disaster risks according to Equation (2).

(1) $$X_{ij}^{s}=\frac{x_{ij}-min\left(x_{ij}\right)}{max\left(x_{ij}\right)-min\left(x_{ij}\right)}\times 100$$

(2) $$X_{i}^{c}=\sum_{j=1}^{4}(X_{ij}^{s}\times w_{j})$$

In Equations (1) and (2), $X_{ij}^{s}$ is the score of the centesimal system in the *j* dimension of disaster-risk perception of resident *i*, where *i* (*i* = 1, 2, ..., 327) represents the individual residents in the sample, *j* (*j* = 1, 2, 3, 4) represents the four dimensions for measuring the disaster-risk perception of rural residents; $X_{i}^{c}$ is the calculated score of the comprehensive perception of disaster risks of resident *i*; $x_{ij}$ represents the factor comprehensive score in the *j* dimension of the disaster-risk perception of resident *i*; $min\left(x_{ij}\right)$ represents the minimum value of the factor comprehensive score in the *j* dimension of disaster-risk perceptions of rural residents;$\;max\left(x_{ij}\right)$ represents the maximum value of the factor comprehensive score in the *j* dimension of disaster-risk perception of rural residents and $w_{j}$ refers to the ratio of the contribution rate of single dimensional variance to the contribution rate of cumulative variance.

(2) Focal variables

The social networks of rural residents were a core independent variable in this study. In the field of sociology research, there are usually two perspectives to discuss social networks. First, the social network is regarded as an analytical tool, with which the relationship between actors and the environment can be clarified [57]. The second is to view the social network as a social structure made up of relationships between actors, and the relationships contained in social networks become the research object [58]. Specifically, in this study, the social network was defined as the collection of nodes (typically referred to as social actors) together with a set of ties (typically known as social relations) that connect pairs of nodes [59], which can provide social support and share risk for people [60]. At present, there is not a recognized research paradigm for the study of social network, and scholars have distinguishing emphases on the study of social network. At the micro level, some scholars explored the structural characteristics of the internal nodes of social networks; some focused on the roles of strong and weak ties [61,62]; and some were concerned about the differences in social relations between different identities (such as kinship, friendship, acquaintanceship, etc.) [63]. Different from the above studies, since the data at each node were limited, the scale of this study was slightly expanded, that is, it considered individuals’ social networks as a whole. In light of the measurement of social capital at the individual level [39,64,65], indicators representing the overall background characteristics of the social network can be selected as the scale, density, heterogeneity, centrality, and quality of the social network. In addition, social capital represents potential resources, which are in the network of personal relationships. In comparison to social capital, the advantage of the social network is that it can express the actual carrier function of social relations. In addition, Borgatti and Li [66] have shown that both ”hard” types of ties (e.g., materials and money flows) and “soft” types of ties (e.g., friendships and sharing-of-information) are crucial (and mutually embedded) in the supply chain context. To sum up, taking into account the background characteristics of social networks as well as their characteristics as carriers, and referring to research on the measurement of social networks by Scherer and Cho [67], Heaney and Israel [60], Reininger, Rahbar, Lee, Chen, Alam, Pope, and Adams [39], and Jones, Faas, Murphy, Tobin, and Mccarty [37], this study categorized residents’ social network variables in terms of background characteristics and carrier characteristics. Further, consider the characteristics of the data obtained, background features are characterized by the scale and heterogeneity of the network, and carrier features are measured by their substance and information transfer functions.

Specifically, Chinese New Year is the most important festival every year for all Chinese people. During the Spring Festival, families get together and also pay New Year greetings to relatives and friends. From the perspective of strong connection and weak connection, “Spring Festival Greeting Networks” reflects the unique manifestation of social networks in China and has been used as a common way of measuring social networks in recent years [68,69]. In view of this, the number of relatives and friends who paid New Year greetings by calling or visiting in the Spring Festival of 2018 was selected to measure the scale of residents’ social networks [34]. Secondly, since the samples selected in this study were rural residents, most of whom were engaged in agricultural activities or types of work other than as teachers, doctors, civil servants, and other public servants in public institutions, the number of public servants among residents’ relatives and friends was selected to measure the heterogeneity of their social networks. In addition, cash gifts for marriage and funerals are an important embodiment of the substance transfer function of social networks in China. Therefore, the frequency of gift expenditure by households in 2018 was used to measure the substance transfer function of residents’ social networks. Finally, the information transfer function was measured via a Likert scale, with 1 representing complete disagreement and 5 representing complete agreement with the statement “You often get disaster-related information from friends and relatives”.

As distinct from flat social networks, another core variable that this study focused on was the degree of trust residents have in community management organizations. In China, the village is the most basic unit of rural society and provides the long-term community in which villagers live and work. As an autonomous form of organization at the grass-roots level in China, villagers’ autonomous committees are responsible for the management of villagers and village-level affairs. Therefore, this study mainly investigated villagers’ trust in their village committee. Referring to research on the definition and measurement of trust by McAllister [70], Luo et al. [71], Lee et al. [72], Ahsan and Dewan [73], Han et al. [74] and Peng, Tan, Lin, and Xu [18], this study designed entries to measure residents’ trust in community management organizations in terms of three dimensions: cognitive trust, emotional trust, and organizational trust. Relating to these, the preconditions for high cognitive trust are reliable performance and excellent technical ability [75,76], which can encourage residents to establish positive cooperative relations with the community and be willing to seek information and help from community management organizations. High-caliber emotional trust is formed from harmonious community relations and friendly interpersonal communication; it reflects the emotional bond between community members and can promote mutual understanding and inclusiveness between residents and community management organizations. Organizational trust is a comprehensive concept that indicates residents’ overall degree of trust in the community management system. It is worth noting that village committees belong to the most basic level of Chinese Government management organizations, so the trust levels of residents in the community management system was evaluated in terms of their degree of trust in the overall governmental system. The specific measures of cognitive trust, emotional trust, and organizational trust are shown in Table 3.

The specific measurement process for trust variables was the same as for the measurement of disaster-risk perception, and therefore will not be repeated here. In the reliability test, Cronbach α values corresponding to cognitive trust, emotional trust, organizational trust, and overall trust levels were all greater than 0.60 (0.71, 0.69, 0.66, and 0.70, respectively), indicating that the entries designed by this paper were internally consistent. In factor analysis, the Kaiser–Meyer–Olkin value corresponding to factor analysis was 0.65, the P value of the Bartlett test for sphericity was 0.000 (less than 0.001), and the cumulative variance contribution rate of the three dimensions was 77.09%, which indicated that the results of the factor analysis were reasonable (see Table 4 for details).

(3) Control variables

Referring to previous studies on the options of control variables (Salvati, Bianchi, Fiorucci, Giostrella, Marchesini, and Guzzetti [15], Devilliers and Maharaj [29], Xu, Qing, Deng, Yong, and Ma [33], Armas [77], Kellens et al. [78]), this paper selected the following as control variables that may correlate with residents’ disaster-risk perception: individual characteristics, family characteristics, community characteristics, and characteristics of disaster experience. Specifically, individual characteristics were represented by gender, age, marital status, duration of residence, and education; family characteristics were described by family population, home address, and annual household income; community characteristics were described in terms of the status of disaster prevention and control in the community and the number of people threatened by disasters in the community; and, disaster experience characteristics were reflected by the number of disasters and the severity of disasters experienced. The definitions of the model variables and the data descriptions are provided in Table 5.

#### 2.3.2. Theoretical Analyses and Research Hypotheses

In terms of social network factors, different characteristics of residents’ social networks may have distinct effects on their perceptions of disaster risks in each dimension. Social networks contain abundant material and information resources. Specifically, for elderly rural residents in mountainous areas, social networks may be an important way to obtain some material or information resources, but are also a crucial channel to obtain social support and security [79]. First, the scale and heterogeneity indicate the background of residents’ social networks; the larger the scale and the stronger the heterogeneity, the more likely residents are to get material and emotional support from the networks. As a result, they may be “fearless”, underestimating the possibility and threat of disasters and overestimating their perceptions of self-efficacy and response efficacy [74,80]. Secondly, although both substance and information transfer functions represent residents’ use of social networks, substance transfer focuses on protection from risks, while information transfer focuses on the prediction of risks. Therefore, although theoretically the effect of the substance transfer function should be same as that of network scale and heterogeneity, the difference is that the more frequently residents transmit disaster-related information (especially information relating to the occurrence of and harm caused by disasters), the more likely they are to have enhanced perception of the possibility and threat of disasters, thereby weakening their self-efficacy and response efficacy evaluations [37,81].

In terms of trust factors, the degree of trust in community management organizations provided a comprehensive evaluation of the long-term performance of the management organizations by residents, concretely reflecting their reliability judgment of the community management organization’s ability to cope with disaster risks. The higher the degree of trust, the more willing residents are to establish a positive cooperative relationship with the community and actively seek information and help from the community management organization [82,83]. At the same time, a high level of trust will form a strong emotional bond within the community, which can provide strong emotional support for residents and thus reduce their fear of disaster risks [84,85]. Therefore, in theory, a high level of trust may reduce residents’ perceptions of the possibility and threat of disasters while increasing their perceptions of the self-efficacy and response efficacy [11,40,86].

According to existing literature conclusions and theoretical analyses, the following hypotheses were proposed for the relationship between rural residents’ social networks, trust, and their disaster-risk perceptions:

**Hypothesis** **1** **(H1).** *There is a significant correlation between the social networks of rural residents in mountainous areas and their disaster-risk perceptions. Specifically:*

**Hypothesis** **1a** **(H1a).** *The scale, heterogeneity, and substance transfer function of rural residents’ social networks are significantly and negatively correlated with their possibility and threat perceptions of disaster risks, significantly and positively correlated with their self-efficacy and response efficacy, and significantly correlated with their comprehensive perceptions—with unclear effect.*

**Hypothesis** **1b** **(H1b).** *The information transfer function of rural residents’ social networks is significantly positively correlated with their possibility and threat perception of disaster risks, significantly and negatively correlated with their self-efficacy and response efficacy, and significantly correlated with the comprehensive perception—with unclear effect.*

**Hypothesis** **2** **(H2).** *There is a significant correlation between rural residents’ trust in community management organizations and their perception of disaster risks. Specifically, cognitive trust, emotional trust, and organizational trust are significantly and negatively correlated with their possibility and threat perceptions of disaster risks, significantly and positively correlated with their self-efficacy and response efficacy, and significantly correlated with their comprehensive perception—with unclear effect.*

#### 2.3.3. The Models

The dependent variables in this study were rural residents’ perception of disaster risks. According to the data types and distribution characteristics of the dependent variables, this study used the Ordinary Least Squares (OLS) regression to control the characteristics of the individual, family, and community, and then gradually added social networks and trust variables to explore their correlations with residents’ perceptions of disaster risks. The model was constructed according to Equation (3):

(3) $$Y_{i}=\beta_{0i}+\beta_{1i}\times Control_{i}+\beta_{2i}\times social\;network_{i}+\beta_{3i}\times trust_{i}+\varepsilon_{i}$$

where $Y_{i}$ refers to the model-dependent variables, specifically including the five indicators of possibility, threat, self-efficacy, response efficacy, and comprehensive perception; $Control_{i}\;$represents the model control variables, including individual characteristics, family characteristics, community characteristics, and characteristics of disaster experience;$\;social\;network_{i}$ represents the model focal variables relating to social network indicators; and $trust_{i}$ represents the model focal variables relating to trust in community management organisations. In addition, $\beta_{0i}$, $\beta_{1i}$, $\beta_{2i}$, and $\beta_{3i}$ are the parameters of the model to be estimated, and $\varepsilon_{i}$ represents model residuals. Analysis of the models in this study was carried out by using Stata 13.0.

## 3. Results

### 3.1. Descriptive Statistics of the Variables

As shown in Table 5, in terms of the dependent variable of residents’ perceptions of disaster risks, the average scores of the four dimensions of possibility, threat, self-efficacy, and response efficacy, as well as comprehensive perception, were 49.81, 60.56, 64.36, 77.94, and 63.70, respectively. Considering these findings as well as the means of the entries for each dimension of disaster-risk perception in Table 1 (the means of the entries for the five dimensions were 3.02, 3.48, 3.82, 4.32, and 3.66, respectively), and taking the mean score of comprehensive perception as the dividing line, the average perception score of the possibility and threat of disaster risks was lower, while the average perception scores of self-efficacy and response efficacy was higher. The reason for this may be that higher evaluations of their self-efficacy and response efficacy reduced residents’ perceptions of the possibility and threat of disaster risk.

In terms of social network variables, the average network scale was 13.39 households; however, the mean value of network heterogeneity was only 1.5 persons. Additionally, the mean of the substance transfer function of social networks was 18.78 times. The mean of the information transfer function of social networks was 3.37, indicating that the frequency of information transfer of social networks was above the intermediate level with a score of 3. Regarding trust variables, the average score of cognitive trust, emotional trust, and organizational trust was 64.63, 62.71, and 73.32, respectively. Considering these findings in combination with the means of entries for each trust variable dimension in Table 3 (the means of the entries for the three aspects were 4.02, 3.97, and 4.37, respectively) indicated that the degree of trust in the management organization was at a higher level.

In terms of individual characteristics, the rural residents were mainly middle-aged, married, and male. Specifically, the average age of the residents was 53.41 years old, 87% were married and 54% were male. In addition, the average education level was 6.29 years and the average duration of residence in the current family was 42.63 years. In terms of family characteristics, 53% of the 327 rural residents believed that their families were in disaster-threatened areas, the average population of the sample families was 4.13, and the average annual household income was 66,185.17 yuan. The annual household income fluctuated greatly, indicating that there were considerable differences among sampled individuals. In terms of community characteristics, the main terrain of all 16 sample villages was mountainous land, and the mean disaster prevention value in communities was 3.89, indicating that most residents believed that some disaster prevention measures had been taken in the community. The average number of people threatened by disasters in communities was 212.65 persons. In terms of the disaster experience of the sampled residents, the average number of disasters experienced by residents was 8.80, but there was large fluctuation in this value. The mean value of the severity evaluation of the disasters experienced was 4.52, indicating that most residents believed that the disasters they had experienced were relatively serious.

### 3.2. Model Results

First, the Spearman rank correlation coefficient was used to test whether there was multiple collinearity between focal variables of the model (Table 6). The results showed that the correlation coefficients between focal variables were far less than 0.8, indicating that there was no serious multicollinearity between focal variables. Secondly, corresponding to the five dimensions of disaster-risk perception—including the possibility, threat, self-efficacy, response efficacy, and comprehensive perception—and considering the role of focal variables in the model, this study constructed 15 multiple linear regression models by gradually adding in the social network and trust variables (Table 7, Table 8 and Table 9). Relating to the dependent-variable indicators, the first model was the estimated result that only incorporated control variables, the second model estimated the result from addition of social network variables to the first model, and the third model estimated the result from addition of trust variables to the second model.

The F test results (Table 7, Table 8 and Table 9) showed that the overall significance of all models was below the 1%, meaning that at least one of the focal variables was significantly correlated with the dependent variables. Comparison of the adjusted R^2^ values of the three models in each dependent variable dimension revealed that, with the exception of the adjusted R^2^ value of the threat perception which decreased with the addition of focal variables, the adjusted R^2^ values of the remaining four dependent variables all significantly increased with the addition of the social network and trust variables. Specifically, for models 1−15, sequentially, the goodness of fit for the probability perception was 5.7%, 6.6%, and 8.0%, respectively; the goodness of fit for the threat perception was 9.0%, 8.6%, and 7.9%, respectively; the goodness of fit for self-efficacy was 12.7%, 13.9%, and 18.8%, respectively; the goodness of fit for response efficacy was 5.3%, 7.2%, and 11.7% respectively; and, the goodness of fit for comprehensive perception was 8.1%, 10.6%, and 15.3%, respectively. Due to the goodness of fit of models 3, 4, 9, 12, and 15, this study focused on the estimation results of these five models and combined these with other models of each dependent variable for the subsequent results analysis.

It can be seen from the estimated results of model 3 (Table 7) that residents’ perceptions of the possibility of disaster risks were significantly and positively correlated with the information transfer function of residents’ social networks (*p* < 0.05) and emotional trust in the community management organization (*p* < 0.01). Specifically speaking, when other conditions remained unchanged, for every one unit increase in the information transfer function of residents’ social networks, their perceptions of the probability of disaster risks increased by 0.111 units, on average; and, for every one unit increase in the emotional trust in the community management organization, their perceptions of the probability of disaster risks increased by 0.149 units, on average. In addition, control variables of gender (*p* < 0.1) and annual household income (*p* < 0.01) both had significant negative impacts on residents’ possibility perceptions of disaster risk, while the home address (*p* < 0.01) had a significant and positive impact on residents’ perceptions of the possibility of disaster risk. In other words, male residents with low annual household income and whose home addresses are threatened by disasters tended to think that disasters were more likely to occur.

Regarding residents’ perceptions of the threat of disaster risks, after adding social network and trust variables on the basis of model 4, which contained only control variables, the goodness of fit of model 6 was reduced; this may have been caused by the insignificant effects of the two focal variables on the threat perception of disaster risk. As the estimation results of model 4 had good goodness of fit for the threat-perception models shown (Table 7), gender (*p* < 0.1), age (*p* < 0.01), education (*p* < 0.01), the number of disasters experienced, and disaster prevention in their communities (*p* < 0.05) were significantly and negatively correlated with the perceived threat of disaster risks. However, the severity of the disasters experienced by the resident (*p* < 0.05) was significantly positively correlated with the perceived threat of disaster risk. The estimation results of model 5 and model 6 (Table 7) revealed the same significant variables as for model 4, but several variables (age, disaster prevention in communities, and severity of experienced disasters) were distinguishing at significant levels. Therefore, on the basis of the estimation results of these three models, it was concluded that male residents who were younger, less educated, and less prepared for disasters in their communities, and had experienced fewer disasters but more severe ones, had higher perceived threat of disaster risk.

In terms of residents’ perceptions of self-efficacy relating to disaster risks, it can be seen from the estimated results of model 9 (Table 8) that cognitive trust (*p* < 0.01) and emotional trust (*p* < 0.01) were significantly and positively correlated with residents’ perceptions of self-efficacy relating to disaster risks, while social networks had no significant effect on their self-efficacy perceptions. To be specific, when other conditions remained unchanged, for every one unit increase in cognitive trust in the community management organization, residents’ perceptions of self-efficacy relating to disaster risks increased by 0.200 units, on average; and, for every one unit increase in emotional trust in the community management organization, their perceptions of self-efficacy relating to disaster risks increased by 0.172 units, on average. It is also worth noting that, according to the estimated results of model 8 (Table 8), the substance transfer function of residents’ social networks was significantly positively correlated with their self-efficacy perceptions (at the 0.1 level). However, this feature was no longer significant after the inclusion of trust variables, which may have been caused by the insufficient explanatory power of the substance transfer function of the social network compared to the impact of trust variables on the self-efficacy of residents. In addition, the control variables of education (*p* < 0.01) and marital status (*p* < 0.1) both had significant and negative impacts on residents’ self-efficacy perceptions relating to disaster risks. In other words, married residents who were more educated tended to think that they were better able to take action to prevent disasters.

In terms of residents’ perceptions of the response efficacy relating to disaster risks, according to the estimated results of model 12 (Table 8), the substance transfer function of residents’ social networks (*p* < 0.05) and organizational trust (*p* < 0.01) were both significantly and positively correlated with the response efficacy for disaster risks. Specifically speaking, when other conditions remained unchanged, for every one unit increase in the substance transfer function of residents’ social networks, their perceptions of response efficacy of disaster risks increased by 0.139 units, on average; and, for every one unit increase in organizational trust in the community management organization, their perceptions of response efficacy relating to disaster risks increased by 0.234 units, on average. In addition, the control variables of the severity of experienced disasters (*p* < 0.01) and the number of experienced disasters (*p* < 0.01) both had significant positive impacts on residents’ perceptions of response efficacy relating to disaster risks. In other words, the more severe and frequent disasters experienced by residents, the stronger their perceptions of response efficacy relating to disaster risks.

In terms of residents’ comprehensive perceptions of disaster risks, according to the estimated results of model 15 (Table 9), the substance transfer function of residents’ social networks (*p* < 0.01), cognitive trust (*p* < 0.1), emotional trust (*p* < 0.01), and organizational trust (*p* < 0.01) were significantly positively correlated with their comprehensive perceptions of disaster risks. Specifically, when other conditions remained unchanged, for every one unit increase in the substance transfer function of residents’ social networks, their comprehensive perceptions of disaster risks increased by 0.157 units, on average; for every one unit increase in cognitive trust, residents’ comprehensive perceptions of disaster risks increased by 0.107 units, on average; for every one unit increase in emotional trust, residents’ comprehensive perceptions of disaster risks increased by 0.204 units, on average; and, for every one unit increase in organizational trust, residents’ comprehensive perceptions of disaster risks increased by 0.112 units, on average. Additionally, control variables of the home address (*p* < 0.01) and the severity of experienced disasters (*p* < 0.01) had significant positive impacts on residents’ comprehensive perceptions of disaster risks, while gender (*p* < 0.01) and annual household income (*p* < 0.1) were significantly negatively correlated with their comprehensive perceptions of disaster risks. In other words, male residents with low annual household income and addresses threatened by disasters, who had experienced more severe disasters, had a higher comprehensive perception of disaster risks.

Combining the findings of all the above models revealed the following. First, the background characteristics of residents’ social networks were not significantly correlated with their disaster-risk perceptions, while the substance and information carrier function of social networks were significantly correlated with some dimensions of disaster-risk perceptions. This suggested that social network variables affecting residents’ disaster-risk perception did not relate to background characteristics but to the use of social networks. Second, compared with social network variables, trust variables were more closely related to the perceived level of disaster risks, which was especially reflected in the correlation with self-efficacy, response efficacy, and comprehensive perception of disaster risks. More specifically, the estimated results of self-efficacy (model 9) showed that trust variables were significantly correlated with self-efficacy perception, while social network variables were not; the estimated results of response efficacy (models 11 and 12) showed that after the addition of trust variables, the effect of substance transfer function on the response efficacy perception decreased in both intensity and significance, and the effect was much smaller than that of the organizational trust variable; the estimated results of comprehensive perception (model 15) showed that the three dimensions of trust variables were significantly correlated with comprehensive perception, while only the social network substance transfer function was significantly correlated with comprehensive perception. The reason for these findings may be due to the response measures of sampled residents to disasters being more concentrated at the public level (such as setting disaster warning boards, planning evacuation routes, etc.), and less at the individual or family level. In this context of public disaster prevention, the degree of trust in management organizations will undoubtedly be more closely related to the level of disaster-risk perceptions. It was worth noting that all of the significant social network variables and trust variables had positive effects on the corresponding dimensions of disaster-risk perceptions, which indicated that trust relating to both the flat social network and pyramidal channels positively affected residents’ perceived levels of disaster risks.

## 4. Discussion

Compared with existing literature, this study made the following marginal contributions. First, previous studies mainly focused on residents’ risk perceptions for single types of disasters, such as earthquakes, floods, and landslides, whereas this study took rural residents threatened by multiple disasters as the research object and measured their perception levels of multiple disaster risks. Second, in existing studies, the measurement of residents’ disaster-risk perceptions mainly considered their understanding and feelings relating to the disaster event itself. This study attempted to evaluate residents’ generalized disaster-risk perception levels from two aspects: their perceptions of the disaster-risk event itself and the degree of mitigation that could be achieved. Third, whereas previous studies did not focus strongly on the impact of social network factors on residents’ perceptions of disaster risks, this study quantitatively explored the correlation between social network factors and residents’ perceptions of disaster risks through the description of background characteristics and carrier characteristics of social networks. Fourth, as distinct from flat social networks, this paper incorporated pyramidal trust channels and empirically explored the correlation between these and residents’ disaster-risk perceptions, as well as further analyzing the different impacts of social network and pyramidal trust factors on residents’ perceptions of disaster risks.

Individual perception of disaster risks is not only the product of individual factors, but also the product of interpersonal and social interactions. In partial support of research hypotheses H1a and H1b, this study found that the information transfer function of social networks was significantly positively correlated with residents’ perceptions of the possibility of disaster risks, and the substance transfer function had significant positive effect on their response efficacy and comprehensive perceptions of disaster risks. These results indicated that the more frequently residents transmitted disaster-related information, especially information relating to the occurrence of and harm caused by disasters, the more their perceptions of the possibility of disaster risks would be enhanced; meanwhile, the substance transfer function of social networks, as an important embodiment of residents’ social support, could enable residents to get material support and security, thus affecting their perceptions of response efficacy. The above results of this paper were consistent with the findings of Iuliana et al. [87], Wu and Li [81] and Jones, Faas, Murphy, Tobin, and Mccarty [37]. For example, Iuliana, ArmaşEugen, and Avram [87] found that the material support residents received could enhance their safety perception levels relating to response measures; Jones, Faas, Murphy, Tobin, and Mccarty [37] found that the more frequent communication among residents, the higher their perception of the possibility of disaster risk. However, the findings of this paper were inconsistent with the findings of Grayscholz et al. [88], in which the background characteristics of social networks (network scale and network heterogeneity) were not significantly correlated with residents’ perceived levels of disaster risks. The reason for this may be that, even though residents had good social network background characteristics, they did not make much use of the corresponding functions of social networks, which weakened the effect of social networks on their perceptions of disaster risks. Furthermore, the background characteristics of social networks represented the potential resources contained in social networks. The carrier characteristics of social networks reflected the mobilized resources. This also suggested that it is not the amount of social relationship resources you have, but the amount of social relationship resources you use that is the key to affect the residents’ perceptions of disaster risks. To sum up, as the carrier of some social resources transmission, social networks can enable residents to obtain practical social securities through its specific functional characteristics, such as information transfer and material support, thus effectively affecting residents’ disaster-risk perceptions. This finding indicates that social networks play an important role in disaster-risk management, which should be more noted.

The current disaster-prevention system in China is mainly community-based disaster prevention [89,90]. In this context, residents’ trust in community management organizations greatly affects their perceptions of disaster risks. In partial support of research hypothesis H2, this study found that cognitive trust and emotional trust had significant positive effects on self-efficacy and comprehensive perceptions of disaster risks, while organizational trust was significantly positively correlated with response efficacy and comprehensive perceptions of disaster risks. The above results of this study were consistent with the findings of ter Huurne and Gutteling [91], Peng, Tan, Lin, and Xu [18] and Han, Wang, and Cui [40], who found that residents’ trust in the public sector was significantly correlated with their perceptions of the controllability of disaster risks. However, inconsistent with research hypothesis H2 and the findings of Fátima and Bernardo [92] and Grayscholz, Haney, and Macquarrie [88], regression estimation results of this study showed that emotional trust was positively correlated with residents’ perceptions of the possibility of disaster risks. This discrepancy in findings may be due to the following reasons. First, emotional trust reflects harmonious community relations and friendly interpersonal communication. The higher the emotional trust, the more frequent the communication between residents will be and the easier it will be to obtain disaster-related information, thus leading to increased perceptions of the possibility of disaster risks. Secondly, the residents in this study were more vulnerable to disasters (the average number of disasters experienced by the sample was 8.80 times). Based on this experience, high emotional trust may lead residents to fear that sudden disasters will harm their cherished communities, thus enhancing their perceptions of the possibility of disaster risks. In addition, it is worth noting that, different to the findings of Bronfman et al. [93] and Han, Xiaoli Lu, Elisa I. Hörhager, and Jubo Yan [74], the empirical results of this study showed that trust factors were not significantly correlated with perceptions of disaster risks. The possible reason is that, although residents believed that community management organizations would take various measures to reduce their losses caused by disasters, the disaster-prone environment still poses a threat to their lives and property. Compared with social network variables, trust variables were more closely related to the perceived level of disaster risk, which was especially reflected in its impact on self-efficacy, response efficacy, and comprehensive perception. This implies that residents paid more attention to the reliability of information and support—the characteristics of pyramid channels—rather than the repeated and uncertain information with high frequency. Furthermore, while information and substance provided by the social network was more convenient and quick, only when the social network implemented its carrier function, it showed close correlation with disaster-risk perception, which also reflected the actual rather than potential support was the vital factor correlating with disaster-risk perception.

In addition, this study found that residents’ individual characteristics (gender, age, education, marital status), family characteristics (home address, annual household income), community characteristics (disaster prevention in the community), and characteristics of disaster experience (the number and severity of experienced disasters) were significantly correlated with different dimensions of the perception of disaster risks. This was consistent with some of the findings of Lindell and Hwang [94], Kellens, Zaalberg, Neutens, Vanneuville, and De Maeyer [78], Xu et al. [95], Ardaya et al. [96], and Tanner and Arvai [97]. For example, Kellens, Zaalberg, Neutens, Vanneuville, and De Maeyer [78] found that individuals’ age, gender, and flood disaster experience significantly affected their perceptions of the threat of flood disaster risks; and Xu, Peng, Su, Liu, Wang, and Chen [95] found that the distance of respondents’ houses from the disaster site and disaster experience were significantly correlated with their perceptions of the possibility of disaster risks. Similarly, the present study found that home addresses that were threatened by disasters significantly affected participants’ perceptions of the possibility of disaster risks, and the age, gender, and disaster experience of participants were all significantly correlated with their perceived levels of the threat of disaster risks.

Although this study provides a useful exploration of the correlations between social networks, trust, and residents’ disaster-risk perceptions, it had some deficiencies. In terms of measuring response efficacy, in consideration of the fact that this study dealt with multiple disasters, the response efficacy specifically referred to the degree to which evacuation could reduce the threat of disasters. However, residents’ perceived effects of different disaster response behaviors might vary, and the perceived effects of other disaster prevention and mitigation measures (such as relocation, reinforcement of houses, etc.) were not considered in this study. In addition, the goal of disaster risk management is to prevent and avoid disasters. Due to the limited space, this study was not extended to include residents’ behavioral responses to disasters. Therefore, future research could explore the differences in residents’ perceptions of response efficacy of different disaster prevention and reduction measures, and the effects of social networks, trust, disaster-risk perceptions, and other factors on residents’ behavioral responses to disasters could be further discussed.

## 5. Conclusions

Based on the empirical analysis and discussion above, this study formed the following main conclusions:

(1) In terms of the characteristics of rural residents’ perceptions of disaster risks, compared with the disaster-risk comprehensive perception scores, participants had lower perception scores relating to possibility and threat and higher perception scores relating to self-efficacy and response efficacy.

(2) The variables of social network that affected residents’ perceptions of disaster risks did not relate to their background characteristics of social networks, but to the use of their carrier characteristics. Specifically, the information transfer function of social networks had a significant positive effect on the perceived level of the possibility; the substance transfer function had a significant positive effect on the perceived level of the response efficacy and comprehensive perception, while the network scale and network heterogeneity had no significant impact on any dimension of disaster-risk perception.

(3) Different dimensions of trust had distinct effects on rural residents’ disaster-risk perceptions. Specifically, emotional trust was significantly and positively correlated with the perception level of the possibility and self-efficacy of disaster risk, cognitive trust was significantly and positively correlated with self-efficacy and the comprehensive perception of disaster risk, and organizational trust was significantly and positively correlated with the perception of response efficacy and the comprehensive perception of disaster risk.

(4) Compared with social network variables, trust was more closely related to the perceived level of disaster risk, which was especially reflected in its impact on self-efficacy, response efficacy, and comprehensive perception.

It is only when residents are aware of the risks they face that they will respond accordingly. Based on the above analysis, in order to improve residents’ perceptions of disaster risks and to strengthen the disaster-risk management ability of communities, this study has the following three suggestions. First, residents’ communication groups or mutual aid groups could be established to strengthen daily contact between community residents and thereby improve residents’ awareness of disaster risks; secondly, strengthening the training of community managers in disaster-related knowledge and organizing disaster prevention and avoidance activities in time to enhance residents’ confidence in dealing with disaster risks could be an effective strategy; and thirdly, combining community disaster prevention with individual disaster prevention, through reasonable guidance, would take advantage of both pyramidal and flat channels in the construction of resilient disaster prevention systems. For example, on the basis of community disaster prevention, community managers can advocate mutual help to strengthen the substantive support between residents, and jointly improve the resilience of residents to confront disaster risks.

## Figures and Tables

**Table 1:** Measurement of disaster-risk perception.

Entry Code	Dimension	Item ^a^	Mean	SD ^b^
P1	Probability	In the next 10 years, there may be disasters near my home.	2.83	1.12
P2		I always feel that disasters will come one day.	3.08	1.32
P3		In recent years, the signs of disasters occurrence have become more and more obvious.	3.17	1.35
T1	Threat	In the next 10 years, if a disaster occurs, your house and land will be damaged.	3.84	1.14
T2		In the next 10 years, if a disaster occurs, your and your family’s lives will be affected.	3.35	1.31
T3		If a disaster occurs, supplies will be cut off.	3.24	1.42
SE1	Self-efficacy	When a disaster occurs, you know the evacuation route.	4.17	1.16
SE2		You know the location of the emergency shelter in the village.	4.00	1.23
SE3		You know the disaster prevention and mitigation measures in the village.	3.28	1.30
RE1	Response efficacy	Evacuation can effectively prevent injury/death.	4.37	0.88
RE2		If I evacuate, I will effectively avoid injury/death.	4.28	0.91
RE3		Evacuation can effectively reduce the emotional and physical pain.	4.33	0.90

Note: ^a^ The Likert scale was used for all entries, with 1 representing complete disagreement and 5 representing complete agreement; ^b^ SD = standard deviation.

**Table 2:** The component matrixes of each risk perception component after rotation.

Items	Component			
	Probability	Threat	Self-Efficacy	Response Efficacy
P1	0.65	0.38	−0.09	0.09
P2	0.82	0.13	−0.22	0.05
P3	0.75	0.09	0.03	−0.11
T1	0.40	0.61	0.08	0.10
T2	0.35	0.72	0.07	0.11
T3	0.01	0.77	−0.09	−0.07
SE1	−0.13	0.19	0.70	0.17
SE2	−0.10	−0.03	0.79	0.07
SE3	0.02	−0.13	0.78	0.09
RE1	0.06	−0.03	0.09	0.86
RE2	0.02	−0.05	0.19	0.86
RE3	−0.07	0.17	0.06	0.81
Eigenvalue	1.96	1.74	1.84	2.21
Explained variance	16.37%	14.48%	15.34%	18.43%
Cumulative variance	16.37%	30.85%	46.19%	64.62%
Cronbach α	0.69	0.63	0.66	0.81

**Table 3:** Measurement of residents’ degree of trust in community management organizations.

Entry Code	Dimension	Item ^a^	Mean	SD ^b^
CT1	Cognitive trust	In the face of future disasters, the community management organization has taken active preparedness measures.	3.92	1.00
CT2		If a disaster occurs, the community management organization will provide information on what to do.	4.12	0.94
AT1	Emotional trust	You are proud to live in this community.	3.82	1.10
AT2		Living in this village will give you more satisfaction than living anywhere else.	4.11	0.92
OT1	Organizational trust	In general, you have faith in government organizations.	4.46	0.84
OT2		People in the community have faith in the decisions of the government.	4.28	0.88

Note: ^a^ The Likert scale was used for all entries, with 1 representing complete disagreement and 5 representing complete agreement; ^b^ SD = standard deviation.

**Table 4:** The component matrixes of each trust component after rotation.

Items	Component		
	Cognitive Trust	Emotional Trust	Organizational Trust
CT1	0.87	0.10	0.10
CT2	0.84	0.11	0.20
AT1	0.14	0.87	0.05
AT2	0.06	0.87	0.14
OT1	0.03	0.13	0.90
OT2	0.36	0.07	0.77
Eigenvalue	1.62	1.54	1.47
Explained variance	26.95%	25.67%	24.47%
Cumulative variance	26.95%	52.62%	77.09%
Cronbach ɑ	0.71	0.69	0.66

**Table 5:** Definition of model variables and data description (*n* = 327).

Category	Variable		Definition and Measure	Mean	Standard Deviation
Dependent variables	Disaster-risk perceptions	Probability	Scores for perception of the possibility of disasters. ^a^	49.81	19.63
		Threat	Scores for perception of the threat of disasters. ^a^	60.56	19.18
		Self-efficacy	Scores for perception of their ability to take action to prevent disasters. ^a^	64.36	21.73
		Response efficacy	Scores for perception of the effectiveness of response measures against disasters. ^a^	77.94	16.75
		Comprehensive perception	Scores for comprehensive perception of disasters. ^a^	63.7	9.62
Focal variables	Social networks	Network scale	The number of households of relatives and friends who visited or called during the Spring Festival of 2018 (households).	13.39	15.84
		Network heterogeneity	The number of relatives and friends working in public institutions (persons).	1.5	3
		Substance transfer function	The number of times of cash gifts given by households in 2018 (times).	18.78	17.28
		Information transfer function	You often get disaster-related information from friends and relatives. ^b^	3.37	1.28
	Trust	Cognitive trust	The degree of cognitive trust in community management organizations. ^a^	64.34	18.01
		Emotional trust	The degree of emotional trust in community management organizations. ^a^	62.71	20.75
		Organizational trust	The degree of overall trust in the management system. ^a^	73.32	18.53
Control variables	Individual characteristics	Gender	Responder’s gender (female = 1, male = 0).	0.46	0.5
		Age	Responder’s age (years old).	53.41	13.5
		Marital status	Responder’s marital status (married = 1, unmarried, widowed or divorced = 0).	0.87	0.35
		Duration of residence	Length of residence of responder (years).	42.63	25.54
		Education	Years of education (years).	6.29	3.7
	Family characteristics	Family population	Family population (persons).	4.13	1.82
		Home address	Is your home address within the disaster threat zone? (yes = 1, no = 0).	0.53	0.50
		Annual household income	Total annual cash income of household (yuan ^c^). Households (yuan ^c^)	66,185.17	72,280.03
	Community characteristics	Disaster prevention	The community has taken some measures to prevent/control disasters. ^b^	3.89	1.08
		Number of people threatened by disasters	The number of people in the community threatened by disasters (persons).	212.65	247.65
	Characteristics of disaster experience	Number of times	The number of times of disaster experience (times).	8.8	12.04
		Severity	In general, how serious are the disasters you have experienced? (Likert scale, not very serious = 1, very serious = 5).	4.52	0.79

Note: ^a^ Centesimal system (0–100); ^b^ Likert scale with 1 representing complete disagreement and 5 representing complete agreement; ^c^ 1 USD = 7.09 yuan (at the time of the study).

**Table 6:** Spearman rank correlation coefficient matrix of focal and control variables in the models.

Variable	1	2	3	4	5	6	7	8	9	10	11	12	13	14	15	16	17	18	19
1	1.000																		
2	0.000	1.000																	
3	0.000	0.000	1.000																
4	0.079	0.058	−0.035	1.000															
5	0.116 **	0.086	−0.139 **	0.346 ***	1.000														
6	0.006	−0.022	0.068	0.018	0.036	1.000													
7	−0.026	0.071	0.039	0.192 ***	0.164 ***	0.042	1.000												
8	−0.047	−0.063	−0.063	0.164 ***	−0.099 *	0.068	−0.015	1.000											
9	−0.132 **	0.095 *	0.197 ***	−0.066	−0.091	0.037	−0.043	−0.208 ***	1.000										
10	−0.053	0.102 *	0.132 **	−0.009	−0.033	0.109 **	0.061	−0.169 ***	0.405 ***	1.000									
11	0.043	0.051	−0.102 *	0.232 ***	0.327 ***	−0.103 *	0.108 *	−0.135 **	−0.494 ***	−0.210 ***	1.000								
12	0.064	−0.034	−0.008	0.130 **	0.099 *	0.027	0.107 *	0.104 *	0.067	−0.014	0.037	1.000							
13	−0.085	−0.236 ***	−0.065	0.132 **	0.118 **	0.027	−0.059	0.156 ***	−0.035	0.046	−0.087	−0.126 **	1.000						
14	0.119 **	−0.104 *	−0.077	0.192 ***	0.054	0.080	0.192 ***	0.084	−0.314 **	−0.180 ***	0.187 ***	0.249 ***	−0.026	1.000					
15	0.156 ***	0.029	0.022	0.262 ***	0.245 ***	0.039	0.166 ***	−0.059	−0.140 **	−0.035	0.246 **	0.051	−0.179 ***	0.314 ***	1.000				
16	0.517 ***	0.071	0.089	0.020	0.080	−0.052	−0.094 *	−0.028	−0.039	−0.141 **	0.086	0.017	−0.036	0.021	0.103 *	1.000			
17	−0.069	−0.029	−0.137 **	0.012	0.124 **	0.060	0.046	−0.025	0.056	−0.033	0.082	0.013	0.051	0.036	0.094 *	−0.119 **	1.000		
18	−0.055	−0.012	0.003	0.073	−0.064	0.160 ***	0.118 **	0.031	0.006	0.009	−0.035	−0.017	0.062	0.033	0.094 *	−0.038	0.149 ***	1.000	
19	−0.015	−0.098 *	0.027	0.071	0.045	0.057	−0.032	−0.005	0.142 **	0.109 **	−0.107 *	0.060	0.196 ***	0.015	0.177 ***	−0.114 **	0.151 ***	0.124 **	1.000

**Note:** *** *p* < 0.01, ** *p* < 0.05, * *p* < 0.1. 1—cognitive trust, 2—emotional trust, 3—organizational trust, 4—network scale, 5—network heterogeneity, 6—substance transfer function of social networks, 7—information transfer function of social networks, 8—gender, 9—age, 10—duration of residence, 11—education, 12—marital status, 13—home address, 14—family population, 15—annual household income, 16—disaster prevention in the community, 17—number of people threatened by disasters in the community, 18—severity of disasters experienced by the respondent, 19—number of times of disasters experienced by the respondent.

**Table 7:** Estimation results of the impact of social networks and trust on rural residents’ possibility and threat perceptions of disaster risks in a multi-disaster environment (standardization coefficient).

Variables	Possibility			Threat		
	Model 1	Model 2	Model 3	Model 4	Model 5	Model 6
Gender	−0.092	−0.094	−0.103 *	−0.099 *	−0.099 *	−0.102 *
	(−1.557)	(−1.587)	(−1.740)	(−1.716)	(−1.682)	(−1.722)
Age	0.048	0.047	0.035	−0.192 ***	−0.194 ***	−0.192 **
	(0.651)	(0.646)	(0.469)	(−2.685)	(−2.700)	(−2.585)
Duration of residence	−0.024	−0.046	−0.057	0.029	0.038	0.041
	(−0.399)	(−0.759)	(−0.942)	(0.490)	(0.628)	(0.670)
Education	−0.062	−0.063	−0.078	−0.199 ***	−0.213 ***	−0.215 ***
	(−0.921)	(−0.911)	(−1.113)	(−3.016)	(−3.090)	(−3.080)
Marital status	−0.024	−0.034	−0.024	0.002	0.002	0.003
	(−0.423)	(−0.582)	(−0.409)	(0.031)	(0.032)	(0.055)
Home address	0.163 ***	0.167 ***	0.197 ***	0.094	0.093	0.092
	(2.810)	(2.879)	(3.321)	(1.639)	(1.628)	(1.549)
Family population	0.009	−0.018	−0.007	0.018	0.032	0.032
	(0.140)	(−0.292)	(−0.111)	(0.288)	(0.510)	(0.508)
Annual household income	−0.141 **	−0.158 **	−0.153 **	−0.079	−0.079	−0.074
	(−2.287)	(−2.524)	(−2.461)	(−1.303)	(−1.275)	(−1.194)
Disaster prevention in the community	−0.088	−0.075	−0.072	−0.126 **	−0.130 **	−0.113 *
	(−1.580)	(−1.348)	(−1.102)	(−2.297)	(−2.356)	(−1.732)
Number of people threatened by disasters in the community	0.024	0.020	0.018	0.051	0.048	0.042
	(0.435)	(0.360)	(0.310)	(0.919)	(0.856)	(0.739)
Severity of disasters experienced	0.082	0.055	0.052	0.133 **	0.149 ***	0.148 ***
	(1.494)	(0.971)	(0.925)	(2.457)	(2.687)	(2.642)
Number of times of disasters experienced	0.000	0.006	0.017	−0.198 ***	−0.199 ***	−0.195 ***
	(0.000)	(0.103)	(0.285)	(−3.445)	(−3.415)	(−3.324)
Network scale		0.030	0.026		−0.037	−0.037
		(0.489)	(0.430)		(−0.609)	(−0.614)
Network heterogeneity		−0.002	−0.013		0.047	0.040
		(−0.035)	(−0.212)		(0.764)	(0.639)
Substance transfer function		0.106 *	0.111 **		−0.071	−0.066
		(1.905)	(2.012)		(−1.295)	(−1.204)
Information transfer function		0.093	0.086		−0.004	−0.002
		(1.637)	(1.519)		(−0.079)	(−0.031)
Cognitive trust			−0.020			−0.025
			(−0.302)			(−0.383)
Emotional trust			0.149 ***			0.013
			(2.646)			(0.227)
Organizational trust			−0.038			−0.049
			(−0.667)			(−0.863)
N	327	327	327	327	327	327
F	2.654 ***	2.439 ***	2.495 ***	3.700 ***	2.905 ***	2.480 ***
R^2^	0.092	0.112	0.134	0.124	0.130	0.133
Adjusted R^2^	0.057	0.066	0.080	0.090	0.086	0.079

**Note:** The values in parentheses indicate the corresponding T values; *** indicates significant at the 1% level, ** indicates significant at the 5% level, and * indicates significant at the 10% level.

**Table 8:** Estimation results of the impact of social networks and trust on rural residents’ self-efficacy and response efficacy of disaster risks in a multi-disaster environment (standardization coefficient).

Variables	Self-Efficacy			Response Efficacy		
	Model 7	Model 8	Model 9	Model 10	Model 11	Model 12
Gender	−0.104 *	−0.099 *	−0.091	−0.036	−0.035	−0.031
	(−1.836)	(−1.742)	(−1.648)	(−0.604)	(−0.591)	(−0.536)
Age	−0.085	−0.086	−0.072	0.042	0.040	0.001
	(−1.206)	(−1.226)	(−1.035)	(0.576)	(0.554)	(0.018)
Duration of residence	0.048	0.030	−0.004	−0.049	−0.060	−0.081
	(0.835)	(0.522)	(−0.076)	(−0.815)	(−0.999)	(−1.356)
Education	0.235 ***	0.243 ***	0.255 ***	0.075	0.088	0.079
	(3.649)	(3.648)	(3.886)	(1.121)	(1.275)	(1.155)
Marital status	0.099 *	0.091	0.094 *	0.072	0.069	0.074
	(1.786)	(1.639)	(1.731)	(1.249)	(1.191)	(1.311)
Home address	0.002	0.005	0.060	0.020	0.017	0.047
	(0.034)	(0.090)	(1.071)	(0.335)	(0.296)	(0.812)
Family population	0.057	0.021	0.027	0.015	−0.016	−0.003
	(0.955)	(0.344)	(0.464)	(0.235)	(−0.261)	(−0.055)
Annual household income	0.076	0.063	0.049	−0.017	−0.020	−0.037
	(1.284)	(1.055)	(0.845)	(−0.280)	(−0.327)	(−0.603)
Disaster prevention in the community	0.135 **	0.152 ***	0.028	0.013	0.036	−0.015
	(2.512)	(2.829)	(0.461)	(0.224)	(0.639)	(−0.226)
Number of people threatened by disasters in the community	−0.009	−0.002	0.009	−0.063	−0.051	−0.020
	(−0.174)	(−0.034)	(0.161)	(−1.110)	(−0.913)	(−0.366)
Severity of disasters experienced	0.038	0.004	0.012	0.258 ***	0.232 ***	0.236 ***
	(0.724)	(0.076)	(0.231)	(4.671)	(4.142)	(4.310)
Number of times of disasters experienced	−0.003	0.003	0.000	0.090	0.109 *	0.1000 *
	(−0.060)	(0.062)	(0.003)	(1.532)	(1.853)	(1.738)
Network scale		0.088	0.081		0.021	0.021
		(1.521)	(1.432)		(0.340)	(0.360)
Network heterogeneity		−0.082	−0.089		−0.110 *	−0.076
		(−1.379)	(−1.534)		(−1.784)	(−1.259)
Substance transfer function		0.077	0.070		−0.020	−0.039
		(1.440)	(1.347)		(−0.364)	(−0.718)
Information transfer function		0.096 *	0.082		0.161 ***	0.139 **
		(1.755)	(1.531)		(2.831)	(2.495)
Cognitive trust			0.200 ***			0.041
			(3.302)			(0.653)
Emotional trust			0.172 ***			0.058
			(3.251)			(1.058)
Organizational trust			0.068			0.234 ***
			(1.293)			(4.241)
N	327	327	327	327	327	327
F	4.969 ***	4.302 ***	4.982 ***	2.507 ***	2.578 ***	3.278 ***
R^2^	0.160	0.182	0.236	0.087	0.117	0.169
Adjusted R^2^	0.127	0.139	0.188	0.053	0.072	0.117

**Note:** The values in parentheses indicate the corresponding T values; *** indicates significant at the 1% level, ** indicates significant at the 5% level, and * indicates significant at the 10% level.

**Table 9:** Estimation results of the impact of social networks and trust on rural residents’ comprehensive perceptions for disaster risks in a multi-disaster environment (standardization coefficient).

Variables	Comprehensive Perception		
	Model 13	Model 14	Model 15
Gender	−0.165 ***	−0.163 ***	−0.163 ***
	(−2.840)	(−2.813)	(−2.875)
Age	−0.086	−0.088	−0.106
	(−1.192)	(−1.244)	(−1.485)
Duration of residence	0.002	−0.021	−0.054
	(0.034)	(−0.348)	(−0.923)
Education	0.043	0.047	0.04
	(0.650)	(0.687)	(0.591)
Marital status	0.077	0.066	0.076
	(1.355)	(1.172)	(1.379)
Home address	0.137 **	0.139 **	0.198 ***
	(2.385)	(2.453)	(3.482)
Family population	0.05	0.008	0.024
	(0.819)	(0.125)	(0.393)
Annual household income	−0.076	−0.093	−0.104 *
	(−1.246)	(−1.519)	(−1.743)
Disaster prevention in the community	−0.023	0.002	−0.08
	(−0.422)	(0.035)	(−1.272)
Number of people threatened by disasters in the community	−0.001	0.005	0.022
	(−0.015)	(0.097)	(0.410)
Severity of disasters experienced	0.25 ***	0.212 ***	0.216 ***
	(4.605)	(3.862)	(4.034)
Number of times of disasters experienced	−0.046	−0.03	−0.029
	(−0.790)	(−0.518)	(−0.511)
Network scale		0.057	0.051
		(0.955)	(0.879)
Network heterogeneity		−0.078	−0.075
		(−1.302)	(−1.260)
Substance transfer function		0.054	0.046
		(0.994)	(0.866)
Information transfer function		0.178 ***	0.157 ***
		(3.185)	(2.872)
Cognitive trust			0.107 *
			(1.723)
Emotional trust			0.204 ***
			(3.772)
Organizational trust			0.112 **
			(2.067)
N	327	327	327
F	3.394 ***	3.428 ***	4.100 ***
R^2^	0.115	0.150	0.202
Adjusted R^2^	0.081	0.106	0.153

**Note:** The values in parentheses indicate the corresponding T values; *** indicates significant at the 1% level, ** indicates significant at the 5% level, and * indicates significant at the 10% level.
